# Assessing cochlear evoked potentials after electrocautery-induced trauma to the utricle

**DOI:** 10.1590/S1808-86942012000200009

**Published:** 2015-10-20

**Authors:** Francisco Carlos Zuma e Maia, Alexandre Dolganov, Luiz Lavinsky

**Affiliations:** a MD, PhD (Hospital de Clínicas de Porto Alegre (HCPA), Universidade Federal do Rio Grande do Sul (UFRGS), Porto Alegre, RS, Brazil); b MD, PhD (Faculdade de Medicina, Pontfcia Universidade Católica do Rio Grande do Sul (PUCRS), Porto Alegre, RS, Brazil); c MD, PhD (Hospital de Clínicas de Porto Alegre (HCPA), Universidade Federal do Rio Grande do Sul (UFRGS), Porto Alegre, RS, Brazil)

**Keywords:** saccule and utricle, surgical procedures, operative, meniere disease

## Abstract

Utriculostomy is a new surgical alternative for Ménière's disease. The basis of this procedure is that the outcome of an electrocautery-induced utricular trauma does not affect cochlear function. However, a demonstration of the hypothesis that this approach to the utricle would preserve hearing is still pending.

**Objective:**

To determine whether any changes would occur in the electrical potentials evoked in the cochlea and auditory nerve before, during, and 1 month after a surgical procedure in the utricule in an animal model.

**Materials and Methods:**

An experimental study. Eight sheep underwent electrocautery-induced utricular trauma, and their cochlear function was assessed by electrocochleography – recording of electrical evoked potentials, in the preoperative, immediate postoperative and medium-term postoperative periods. The results were analyzed statistically.

**Results:**

There were no statistically significant variations in amplitude (*p* = 0.099) and latency (*p* = 0.591) before and 1 month after the surgical procedure. There was a statistically significant change in the summation of the potential/action potential area ratio (*p* = 0.0122), a calculated loss of 11.8 dB.

**Conclusion:**

The intervention performed in this study enabled us to conclude that, taking into account the impaired electrophysiological responses observed during and 1 month after the surgical procedure, hearing was preserved in the operated sheep.

## INTRODUCTION

Since 1878, when Kessel mobilized the stapes in cases of otosclerosis for hearing recovery, otologists have engaged in research to develop surgical techniques that enable the approach to the labyrinth (otic capsule and membranous labyrinth) for recovery of the auditory function and removal of the agent causing organ dysfunction or the patient's clinical symptoms (hypoacusis, vertigo and tinnitus)^1^.

Surgeons, however, were aware that such interventions could damage labyrinthine structures extremely sensitive to vibration, as well as endolymph flow, and tried to avoid a surgical approach to the inner ear, acting fairly distant from the cochlea.

Historically, there is a considerable literature on surgical procedures proposed for the treatment of vertigo, according to the underlying disease^2^. In Ménière's disease, for example, some of the techniques used act on the distal or proximal endolymphatic system^3^.

A new surgical alternative for Ménière's disease, which is still in the experimental stage, is the method proposed by Lavinsky et al.^4^, the so-called utriculostomy, which consists in obtaining a permanent fistula in the membranous labyrinth, so as to establish communication between the endolymphatic and perilymphatic spaces at the utricle level. The method was performed in sheep through the oval window, using a radiofrequency microcautery with a timer to control temperature and exposure time. Since this method does not act on the saccule, which is intimately related to the anatomical and functional unit, the cochlea, the authors expect to demonstrate that the utriculostomy will produce less cochlear repercussion than sacculotomies, which generate a high incidence of neurosensory hypoacusis.

In the study by Lavinsky et al.^4^, the reconstitution of a fragile new membrane was histologically demonstrated, which would probably play a role as a discharge “valve” in situations in which hydrops may occur. However, the demonstration of the hypothesis that the approach to the utricle would preserve hearing is still pending.

Based on the operational hypothesis that the electrocautery-induced utricular trauma would not affect hearing, the objective of this study was to determine whether any changes would occur in the electrical potentials evoked in the cochlea and auditory nerve in sheep.

## MATERIAL AND METHODS

This experimental study was conducted in the Animal Experiment Unit at our institution, in accordance with the international procedures and regulating rules of research involving living animals.

Sample size calculation was determined based on a pilot study including four animals, in which the paired-sample Student *t* test was used in the comparison between preoperative and immediate postoperative means, as well as in the evaluation of the preoperative and medium-term (1 month) postoperative periods with magnitude of effect of two standard deviations (SD), α = 0.05, and 90% power.

Exclusion criteria were: due to anatomical reasons of the external acoustic meatus, the test and surgical procedure could not be performed; the animal died during the experimental study period. The animals which showed normal cochlear potentials before the surgical procedure were included in the study.

Eight female Texel sheep, with 40 kg mean live weight and aged around 18 months, were submitted to the assessment of cochlear evoked potentials before, during and 4 weeks after electrocautery-induced utricular trauma. The animals were not killed and were taken to their place of origin as soon as possible after the end of the experiment. One animal was excluded from the study due to death from unknown cause 25 days after the surgical procedure.

The anesthetic protocol was the same used by Lavinsky et al.^4^.

All surgical procedures were performed by the same surgeon (adviser in the present study).

### Electrocautery-induced utricular trauma

**Figure 1:**
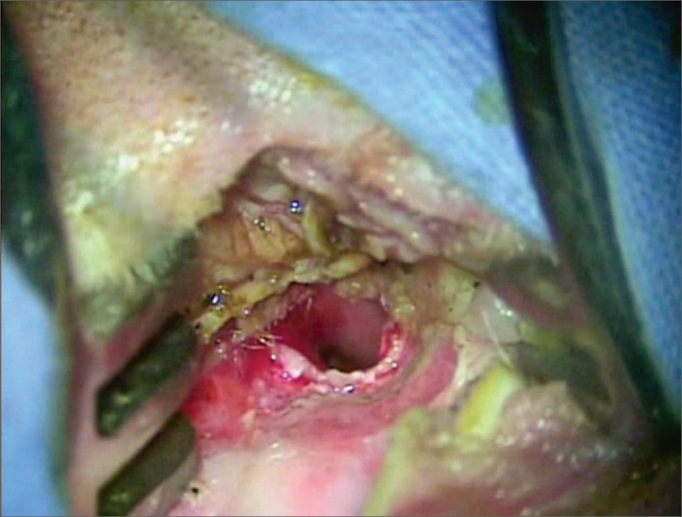
Endaural approach.

**Figure 2:**
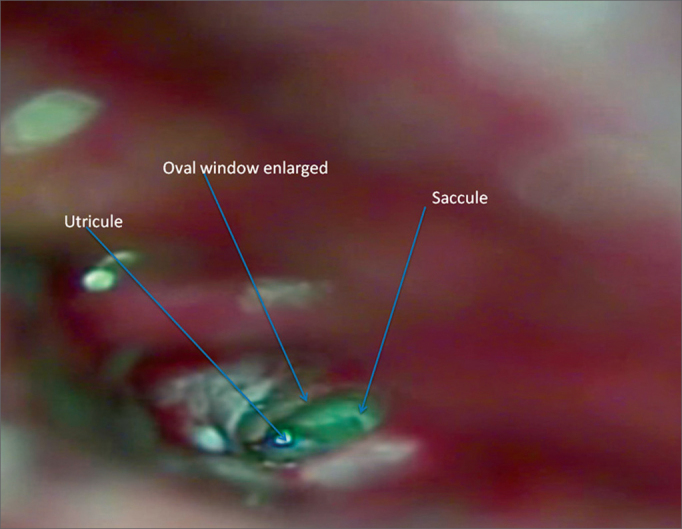
Oval window enlarged, utricle and saccule.

The promontory, the oval and round windows, the facial nerve, the chorda tympani, the pyramidal apophysis, and the tensor ligament of the stapes were identified after endaural approach (Figure 1). The tensor ligament of the stapes was then sectioned and the incudostapedial joint was disarticulated. The stapes suprastructure was fractured, and all of the platina was removed. With the diamond drill, approximately 2 mm of the posterior rim of the oval window was removed toward the facial nerve, thus enlarging the oval window and facilitating the cauterization procedure in the utricle. (Figure 2).

The anterior wall of the utricle, which is partially visible during otomicroscopy, was cauterized using the Lavinsky/HCPA otologic microthermocautery with a 0.2-mm pointer for 0.5 seconds at intensity of 3.5 W. The cauterization was repeated in three locations, close to one another.

The oval window was closed with adipose tissue taken from the region next to the endoperiauricular incision and then covered with Gelfoam®. The tympanum was restored and the meatus was packed with Gelfoam®. Endoperiauricular incision was sutured with 3.0 mono nylon simple stitches. The region was finally covered with a compressive dressing. The animals received antibiotic prophylaxis with sodium penicillin (40 mg/kg/day) 1 day before and up to 3 days after surgery.

Postoperative protocol involved visual observation of the presence or absence of nystagmus, as well as alterations in static and dynamic balance, in the immediate postoperative period (2, 4 and 8 hours after surgery); after 24 hours, additional observations were performed regarding the animal's general status and the presence or absence of nystagmus and alterations in balance.

### Recording of auditory evoked potentials

A tone burst stimulus was delivered at the external acoustic meatus in frequencies ranging between 4200–6820 Hz and intensity of 82 SPL, according to the protocol of electrophysiological threshold recording of normal cochlear potentials in sheep^5^.

Summating potential (SP) and action potential (AP) areas were calculated using the Riemann integral.

To measure the degree of procedure-related hearing loss and to estimate the new hearing threshold, we used the equation to calculate the equivalent sound level (ESL) (*L*_*eq*_)^6^.

### Hearing progress analysis

The animals returned for a new hearing measurement 1 month after the surgical intervention, for recording of auditory evoked potentials, with the same anesthetic protocol used in the first part of the experiment. After completion of the study, the animals were not killed, returning to the breeder's/supplier's farm.

### Statistical analysis

The variables amplitude and latency were compared at the time points before, during and after procedure using the analysis of variance of repeated measurements (general linear model), with Bonferroni post hoc test. The corresponding nonparametric test was used for the variable area: the Friedman test. The Wilcoxon test was used to find differences by comparing two experimental time points.

All tests performed were two-tailed, and *p* values less than or equal to 0.05 were considered to be statistically significant. Statistical analysis was performed using the Statistical Package for Social Sciences (SPSS) 10.0 for Windows.

## RESULTS

All animals behaved in a docile way during the preoperative period (anesthetic induction) and immediate postoperative period. There were no cases of bleeding; only one death from unknown cause 25 days after the surgical procedure, during confinement in the breeder's farm.

**Figure 3:**
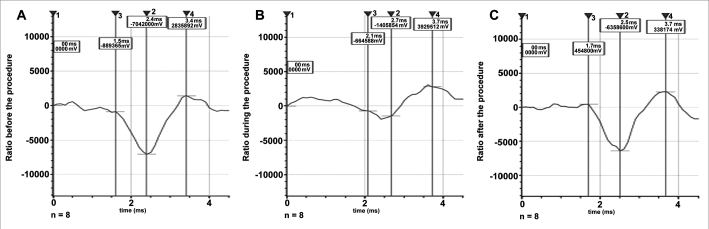
Median values of cochlear evoked potentials before (A), during (B) and after (C) the procedure.

Eight sheep were studied, with measurement of amplitude, latency and SP/AP area ratio before, during and after the procedure (Table 1 and 2, Figure 3).

**Table 1:** Results of the recording of cochlear evoked potentials in 8 sheep with measurements of amplitude, latency and SP/AP area before, during and after the procedure.

Sheep		Amplitude (μV)			Latency (ms)			SP/AP Area (μV)	
	Before	During	After	Before	During	After	Before	During	After
1	5760000000	2592000000	4704200000	3400000	3800000	3700000	11794099023	10002000000	5929133301
2	8080000000	5696000000	4393533237	4300000	5900000	4100000	9172200195	1931700195	3196447266
3	9728000000	3854000000	5385641113	3700000	5104000	3930000	15042200195	10075000000	4990200195
4	6334000000	3176580000	6718000000	4130000	4480000	3600000	11677600586	16007830078	9571600586
5	6400000000	6177000000	6510000000	2500000	4400000	3600000	14813600281	10365706055	9773200195
6	8190000000	9854000000	6542000000	2766000	3960000	3700000	8437400391	1775000000	4432000488
7	6334000000	5300340000	5360000000	2930000	4600000	3250000	11677600586	1107830078	1012799805
8	7200000000	6176000000	6385600098	3810000	4120000	3840000	11813600434	1065706055	6608400391

**Table 2:** General data from the study population

Variable	Before	During	After
Amplitude (μV)	7.253 ± 1.328	5.35 ± 2.27	5.75 ± 0.90
Latency (ms)	3.442 ± 0.65	4.545 ± 0.63	3.715 ± 0.25
Area (μV*ms)	11.7	5.9	5.460
	(9.80–14.1)	(1.3–10.3)	(3.50–8.83)

n = 8, mean. ± standard deviation, median (interquartile range).

The analysis of variance of repeated measurements revealed statistically significant differences in the comparison of latency (*p* = 0.011), but not in that of amplitude (*p* = 0.099). A difference was also found in the comparison of areas, through the Friedman's test (*p* = 0.012).

**Figure 4:**
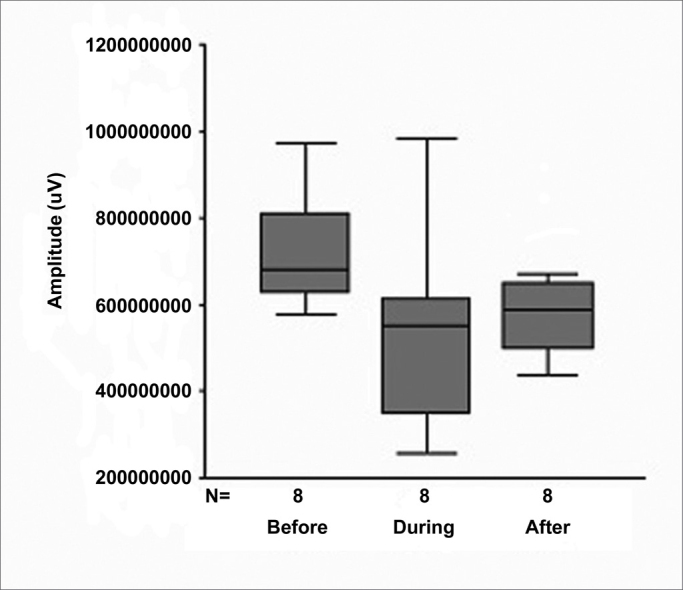
Box plot graph of amplitude at the three experimental time points.

Figure 4 shows the behavior of amplitude at the three experimental time points.

Table 3 shows Bonferroni test p value for the variable latency.

**Table 3:** Bonferroni test for the variable latency.

Latency (ms)	Difference in mean	*p* value
Before vs. during	−1.103	0.006
During vs. after	−0.830	0.017
Before vs. after	−0.273	0.591

n = 8, mean ± standard deviation.

**Figure 5:**
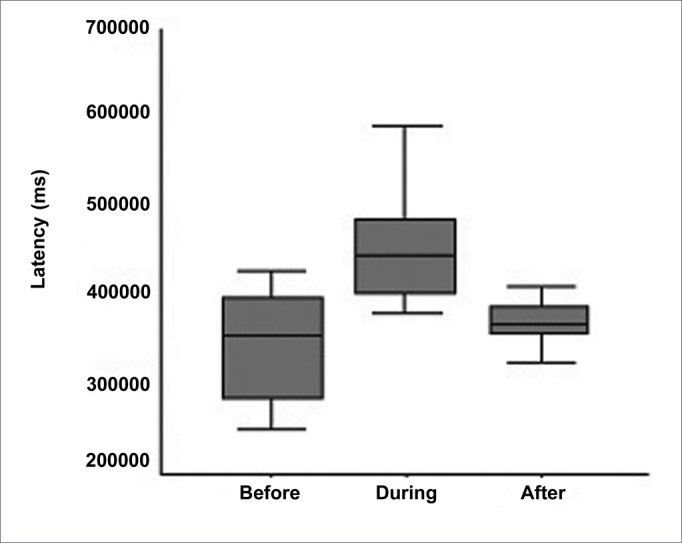
Box plot graph of latency at the three experimental time points.

Figure 5 shows the behavior of latency at the three experimental time points.

Table 4 shows the comparisons between the variable area at the experimental time points: before vs. after; before vs. during; and during vs. after procedure (by Wilcoxon test).

**Table 4:** Comparison of differences in area by Wilcoxon test.

Area (μV^*^ms)	Difference in median	*p* value
Before vs. during	5.262	0.0252
During vs. after	0.852	0.5752
Before vs. after	−6.110	0.0122

**Figure 6:**
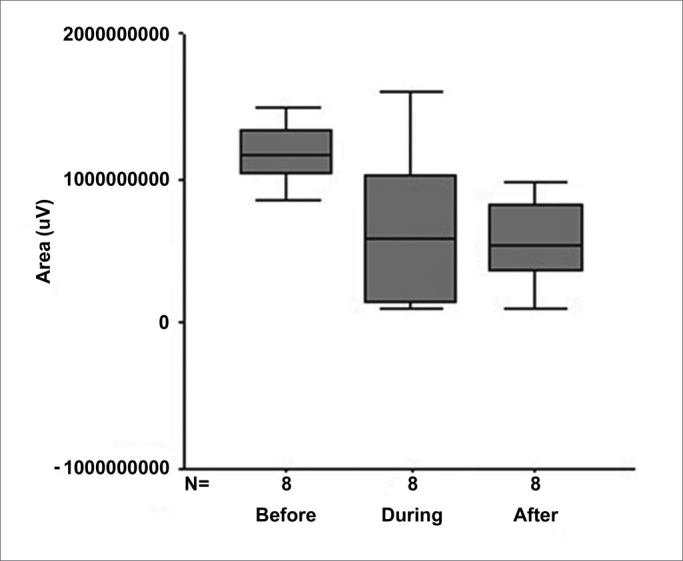
Box plot graph of area at the three experimental time points.

Figure 6 shows the behavior of area at the three experimental time points.

Loss of intensity in the physiological response to the initial stimulus of 82 dB after the procedure was 11.8 dB, estimated from the calculation of ESL: Log (4.200–6.820 × 2 × 82) = 5.95 corresponding to the mean SP/AP area ratio of 11.7 μV^*^ms; 30 days after the procedure the mean area was 5.460 μV^*^ms, with ESL estimated at 2.78, which, once the value of auditory stimulus intensity was recalculated, resulted in 70.2 dB.

In the immediate postoperative period and 24 hours after surgery, we observed in all animals the presence of horizontal nystagmus rotating counterclockwise to the operated ear, as well as alterations in static and dynamic balance. All animals remained with ipsilateral inclination of the head toward the side of the operated ear for at least 30 days after the surgical procedure.

## DISCUSSION

Normal cochlear and vestibular function depends on the integrity of the inner ear, with a physical separation of endolymphatic and perilymphatic compartments by the membranous labyrinth. Disorders occurring between these relationships may lead to a deep loss of function. Therefore, surgical entry into the inner ear has been traditionally avoided.

Recent advances in neurotologic surgery in animal models have challenged the traditional belief that violating the labyrinth is incompatible with hearing^7^.

Endolymphatic shunt procedures, such as sacculotomies (Tack operation) and cochleosacculotomy, a surgery described as a simple technique, effective in the treatment of vertigo in patients with Ménière's disease in whom medical therapy has failed, have been abandoned by some authors who observed significant long-term worsening of hearing thresholds in these patients^8^.

The present study aims to establish the safety of utriculostomy regarding hearing loss. In order to maximize the confidence level of this decision, we opted to produce a relevant injury to the utricle so that, if hearing was preserved, safety regarding utriculostomy could be well grounded, since this procedure is much more conservative, in terms of aggressiveness, to the utricular structure.

As in other studies^9^, sheep was an appropriate animal model for experimental surgery in otology. The electrocautery-induced utricular trauma was confirmed by the presence of nystagmus and alterations in static and dynamic balance for 24 hours, as well as by the ipsilateral inclination of the head toward the side of the operated ear for at least 30 days, which are signs indicative of utricular trauma, as observed in the postoperative protocol.

The analysis of cochlear action potential parameters, representing the most important potential obtained by transtympanic electrocochleography and reflecting the sum of the responses of synchronous discharges of thousands of individual nerve fibers, which are limited to high-frequency bands, showed that:

1. There were no statistically significant alterations in the parameter *amplitude* when comparing the results before, during and after 1 month (*p* = 0.099).
2. In the parameter *latency*, there was alteration during the procedure, but with recovery after 1 month (*p* = 0.591), revealing that wave propagation time in the cochlea increased during the surgical procedure, with recovery after 1 month. Therefore, we may infer that internal ciliated cells of the cochlea and the auditory nerve were not extensively compromised, which was demonstrated when we associated this result with studies in guineas pigs^10^, in which the action of acute excytotoxicity was shown to cause a rupture in the ending of the afferent dendrite, and only one part of the postsynaptic membrane remains attached to the presynaptic. One day after the trauma, the afferent dendrite begins to grow, some filopodia reach the internal ciliated cell, and recovery of cochlear potential starts. Five days after the trauma, normal synaptic pattern can be observed again, resulting in complete functional recovery, evidenced by the normalization of cochlear action potential. The present study establishes, based on the results, that the neurosensory damage caused by the procedure was small, bearing in mind that nerve regeneration occurred after 30 days, as demonstrated by cochlear potentials.
3. The analysis of the variable *area* was included in our study since it also represents a good parameter of interpretation of nerve conduction studies. The calculation of the SP/AP area ratio, described in some studies^11^, ^12^ and used in the present study, stands out as a very sensitive test to assess cochlear response to hearing stimulus in Ménière's disease, as well as in situations in which the cochlea is at risk due to surgical injury^13^.

Since SP/AP area ratio is the integral of a function of the combination between amplitude and duration of action potential, Riemann integral reflects the number and synchronism of activated nerve fibers. In our study, there was a statistically significant decrease in the SP/AP area ratio (*p* = 0.0122) during the procedure, which remained after 1 month.

This decrease in the number and synchronism of activated nerve fibers may originate from some inflammatory process resulting from surgical trauma, as demonstrated in the histological study of injuries to the vestibule caused by surgery of the inner ear in guinea pigs^14^, as well as from a secondary neuronal degeneration process occurring through destructive cascades activated during glutamate-mediated excytotoxicity^15^. Most of these long-suffering neurons manage to recover and reestablish synapse with sensory cells, whereas others, due to electrophysiological alteration resulting from calcium homeostasis, ultimately end in apoptosis.

It is important to point out that the ESL, defined as a continuous noise level that has the same acoustic energy of initial oscillatory levels for a certain period of time^6^, and its calculation is used for that purpose, allowed us to define as 11.8 dB the loss of intensity in the physiological response to the initial stimulus. Thus, stimulus intensity, which prior to the surgical procedure was of 82 dB, after 1 month fell to 70.2 dB, expressed in this study as the reduction in amplitude of the SP/AP area ratio and increase in latency, indicating that, regardless of impaired electrophysiological responses, there was preservation of hearing as a sensory function.

The association between utricular surgery and hearing preservation is found only in two studies in the literature^16^, ^17^. By describing interventions in utricular maculae through the oval window using argon laser irradiation in guinea pigs^16^ and CO_2_ and KTP laser irradiation in cats^17^, the authors show that, in spite of the histological evidence of trauma and total degeneration of the sensory epithelium, in all animals hearing was preserved, which, in accordance with the results of the present study, confirms the hypothesis that: the utriculostomy proposed by Lavinsky would have less auditory repercussion.

Previous experiments have suggested that the mechanisms of hearing loss after injury to the inner ear involve factors other than only loss of ciliated cells^16^. Thus, surgical factors that affect hearing preservation or loss are difficult to identify. One might speculate that more-experienced surgeons will perform more precise, and involuntarily less traumatic, lesions. In our experiment, performed by the same surgeon with experience in this technique performed in a previous study^11^, we demonstrated that the sheep utricle is capable of withstanding surgical trauma without complete loss of cochlear function.

## CONCLUSION

Surgery of the inner ear with hearing preservation imposes an important challenge on neurotologic procedures. Ability to perform selective procedures in the vestibular organ enhances the potential to develop new procedures for the treatment of disabling labyrinthopathies.

The intervention in the utricle performed in this study enabled us to conclude that, taking into account the impaired electrophysiological responses observed during and 1 month after the surgical procedure, there was hearing preservation in the operated sheep.
